# Body mass index, diet, physical inactivity, and the incidence of dementia in 1 million UK women

**DOI:** 10.1212/WNL.0000000000008779

**Published:** 2020-01-14

**Authors:** Sarah Floud, Rachel F. Simpson, Angela Balkwill, Anna Brown, Adrian Goodill, John Gallacher, Cathie Sudlow, Phillip Harris, Albert Hofman, Sarah Parish, Gillian K. Reeves, Jane Green, Richard Peto, Valerie Beral

**Affiliations:** From the Cancer Epidemiology Unit (S.F., R.F.S., A.B., A.B., A.G., G.K.R., J.G., V.B.) and MRC Population Health Research Unit (S.P.), Nuffield Department of Population Health (R.P.), and Department of Psychiatry (J.G.), University of Oxford; Centre for Medical Informatics (C.S.), Usher Institute of Population Health Sciences and Informatics, University of Edinburgh, UK; Royal Prince Alfred Hospital (P.H.), Sydney, Australia; and Department of Epidemiology (A.H.), Harvard T.H. Chan School of Public Health, Boston, MA.

## Abstract

**Objective:**

To help determine whether midlife obesity is a cause of dementia and whether low body mass index (BMI), low caloric intake, and physical inactivity are causes or merely consequences of the gradual onset of dementia by recording these factors early in a large 20-year prospective study and relating them to dementia detection rates separately during follow-up periods of <5, 5 to 9, 10 to 14, and 15+ years.

**Methods:**

A total of 1,136,846 UK women, mean age 56 (SD 5) years, were recruited in 1996 to 2001 and asked about height, weight, caloric intake, and inactivity. They were followed up until 2017 by electronic linkage to National Health Service records, detecting hospital admissions with mention of dementia. Cox regression yielded adjusted rate ratios (RRs) for first dementia detection during particular follow-up periods.

**Results:**

Fifteen years after the baseline survey, only 1% were lost to follow-up, and 89% remained alive with no detected dementia, of whom 18,695 had dementia detected later, at a mean age of 77 (SD 4) years. Dementia detection during years 15+ was associated with baseline obesity (BMI 30+ vs 20–24 kg/m^2^: RR 1.21, 95% confidence interval 1.16–1.26, *p* < 0.0001) but not clearly with low BMI, low caloric intake, or inactivity at baseline. The latter 3 factors were associated with increased dementia rates during the first decade, but these associations weakened substantially over time, approaching null after 15 years.

**Conclusions:**

Midlife obesity may well be a cause of dementia. In contrast, behavioral changes due to preclinical disease could largely or wholly account for associations of low BMI, low caloric intake, and inactivity with dementia detection during the first decade of follow-up.


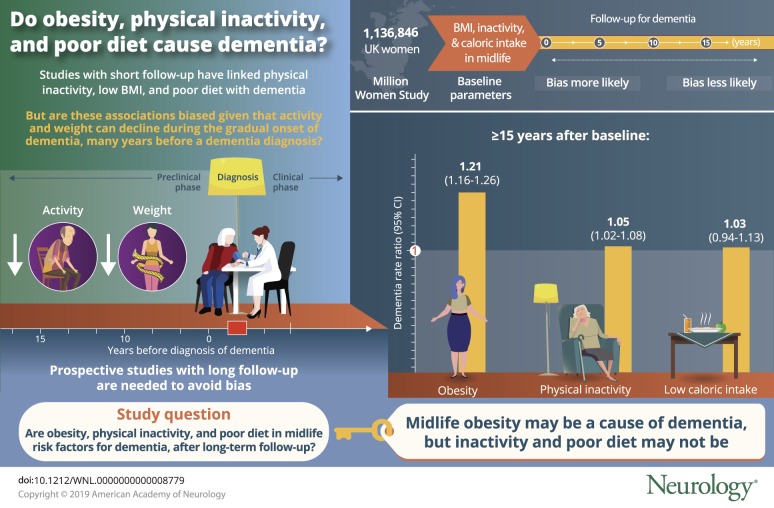


The pathologic processes that eventually culminate in dementia may have slowly increasing preclinical cognitive effects for a decade or more before there is a definite clinical diagnosis. During this period, preclinical disease can gradually affect behavior, reducing mental and physical activity, changing dietary and other habits, reducing caloric intake, and causing appreciable weight loss. Hence, for at least a decade before people are diagnosed clinically with dementia, there are, on average if not in each individual, appreciable reductions in physical activity and body mass index (BMI).^1–4^ Thus, preclinical disease can, by behavioral or other means, cause weight loss.

In the general population of people who have not, or not yet, been diagnosed as having dementia, low BMI is therefore associated with the probability of dementia being diagnosed over the next 5 or 10 years.^5,6^ Because this association might, however, be due merely to the process of reverse causality described above, the existence of the association cannot be used straightforwardly to determine whether, as well as preclinical disease causing weight loss, low BMI somewhat hastens the onset of dementia.

To address this question, prospective studies are needed that last much longer than the period over which the effects of reverse causality are likely to be appreciable. Within such studies, the biases due to the effects of reverse causality would be expected to be much stronger during the first 10 (or, particularly, the first 5) years of follow-up after BMI was measured than during the second decade of follow-up. However, in the absence of the onset of any of the various diseases that affect weight, BMI in middle age correlates well with BMI 20 years later.^7^ Hence, if low BMI (or obesity) actually causes or aggravates dementia, then reasonably unbiased evidence of this may emerge only during the second decade of follow-up, after the main short-term effects of reverse causality have faded. Two recent meta-analyses have presented evidence that reverse causality could well account for the short-term inverse associations of BMI with dementia but that on longer follow-up obesity (BMI >30 kg/m^2^) is positively associated with the incidence of dementia; however, they included only limited numbers of cases arising >15 years after BMI was measured.^5,6^ Other meta-analyses,^8–10^ also dominated by findings during just a few years of follow-up, have reported associations of poor diet and physical inactivity with the incidence of dementia, but these findings could well be largely or wholly due to reverse causality; consistent with this, a meta-analysis^8^ noted that the effects of physical inactivity seemed to weaken with longer follow-up.

To minimize biases due to reverse causality, the first few years of follow-up are often excluded in meta-analyses of prospective study data, as are the participants who already had some chronic disease when the baseline measurements were recorded. For example, many types of cancer can cause weight loss some time before diagnosis, so investigations of causal relationships between BMI and cancer commonly exclude the first 5 years of follow-up.^11^ Dementia usually has a more insidious onset than cancer, so bias due to reverse causality may well last longer.

The aim of the present report is to help determine whether midlife obesity is a cause of dementia and whether low BMI, low caloric intake (in women who had not recently changed their diet because of disease), and physical inactivity are causes or merely consequences of the gradual onset of dementia. These factors were recorded at or near the start of the Million Women Study, and we relate these recorded values to dementia detection rates during the separate follow-up periods of <5, 5 to 9, 10 to 14, and 15+ years later.

## Methods

### Study population

The Million Women Study is a population-based prospective study.^12^ In 1996 to 2001, women invited for National Health Service (NHS) breast cancer screening at 66 screening centers in England and Scotland were asked to join the study by completing the recruitment postal study questionnaire. This sought information on health, sociodemographic, anthropometric, and lifestyle factors. Resurvey questionnaires are posted to study participants every 3 to 5 years to assess changes in these factors and to inquire about other exposures. Questionnaires can be viewed at millionwomenstudy.org.

### Standard protocol approvals, registrations, and patient consents

Ethics approval was granted by the Oxford-Anglia Multi-Centre Research Ethics Committee. All participants gave written consent for follow-up.

### Measures

Height, weight, and physical activity were reported at recruitment. The present analyses are restricted to those with a height of 1.2 to 2.0 m and weight of 35 to 150 kg, from which (self-reported) BMI was calculated as weight divided by the square of height. BMI <20 kg/m^2^ is described as low, 20 to 24.9 kg/m^2^ as desirable, 25 to 29.9 kg/m^2^ as overweight, and ≥30 kg/m^2^ as obese. In this cohort, BMI at recruitment is an excellent correlate of measured BMI a decade later because, in a validation study in which weight and height were measured in a sample of the cohort about a decade after recruitment, there was a 95% correlation between the baseline BMI based on self-reported height and weight and the BMI based on measured height and weight.^7^

Participants were asked at recruitment about exercise strenuous enough to cause sweating or a fast heartbeat, and ≈90% were also asked a second multiple-choice question to help assess inactivity: “About how often do you do any exercise?” Only the question about any exercise is used in these analyses, classifying women as inactive (rarely/never or <1 time per week) or active (≥1 time per week). Three years after recruitment, 589,896 of these women reported hours per week of housework, walking, gardening, cycling (rare), and strenuous activity (also rare), and the mean excess energy consumption (in metabolic equivalent of task–hours) resulting from all such activities was 20% lower among those who had been classified as inactive than those classified as active at recruitment.^13^ The present analyses are restricted to the 1,136,846 women who, at recruitment, had been asked the question about inactivity, had reported their height and weight within the acceptable ranges, and were 48 to 65 years of age, which was at that time the range for routine breast screening invitations (data available from Dryad, additional Methods 1, doi.org/10.5061/dryad.3s755j8).

To investigate the association of dementia with low caloric intake, we used information on some 130 quantitative and semiquantitative dietary items^12^ sought 3 years after recruitment (data available from Dryad, additional Methods 2, doi.org/10.5061/dryad.3s755j8). After the exclusion of 409,536 nonrespondents, 113,666 respondents who had changed their diet during the previous 5 years due to illness, and 3,450 whose replies suggested a caloric intake outside the range of 500 to 3,500 kcal/d, 610,069 women remained, among whom this assessment of caloric intake was analyzed in relation to subsequent dementia detection rates (data available from dryad, additional Methods 1). Caloric intake was divided into 5 similar-sized groups, and comparisons were made across these groups and between the lowest and the other 4 groups. Secondary analyses examined other indices of poor diet in these 610,069 individuals, including low protein intake and an index of Mediterranean diet. There was good short-term repeatability for most dietary questions,^14^ and intakes did not change appreciably over time in the cohort as a whole.^15^

All participants were registered with the NHS. With the use of each individual's unique NHS number and date of birth, study participants were linked electronically to routinely collected NHS data on hospital admissions (as day cases or as inpatients), deaths, and emigrations by NHS Digital in England (in which up to 20 diagnoses are coded for every hospital admission)^16^ and by the NHS Central Register and Information Services Division in Scotland (in which up to 6 diagnoses are coded for every hospital admission).^17^ Hospital diagnoses are coded according to the World Health Organization's ICD-10.^18^ The main outcome in these analyses is the first mention, in any hospital record, of dementia (ICD-10 code F00-F03 or G30), excluding the 26 women with such a record before recruitment. Some analyses also examined associations separately for women whose first hospital record of dementia attributed it to Alzheimer disease (F00 or G30), vascular dementia (F01), or dementia type unspecified (F03); there were far fewer cases of each of the other specific types of dementia (F02). The relatively small number of deaths with dementia on the death certificate but not in any hospital record were not included in the main analyses but were included in sensitivity analyses.

Seventy six thousand three hundred and one of the included Million Women Study participants have also been linked to the UK Clinical Practice Research Datalink (CPRD), which by 2017 included primary care records for 8% of the UK population.^19,20^ Because not all women with a primary care record that mentions dementia will have had, or will soon have, a hospital record that mentions dementia, we investigated the probability of first admission to hospital with any mention of dementia by time since the first mention of dementia in primary care (data available from Dryad, additional Methods 3, doi.org/10.5061/dryad.3s755j8).

### Statistical analyses

After the exclusions described above (data available from Dryad, additional Methods 1, doi.org/10.5061/dryad.3s755j8), women contributed person-years from the exact questionnaire completion date until whichever date came first of the following: death, first hospital record of dementia, NHS registration ending (≈1%, mostly emigrants from the United Kingdom), and December 31, 2017, in England or March 31, 2017, in Scotland.

Cox regression was used to estimate the dementia detection hazard ratios (hereafter called rate ratios [RRs]) and 95% confidence intervals (CIs) associated with low BMI, obesity, low caloric intake, and physical inactivity within each of 4 follow-up periods (<5, 5–9, 10–14, 15+ years). Time from baseline was the underlying time variable. To ensure that analyses compared dementia detection rates in women who were as similar as possible in all other respects, all analyses were routinely stratified by single year of birth (≤1930, 1931…1949, 1950 and later) and by calendar year at recruitment (1996, 1997, 1998, 1999, 2000, 2001 and later) or, for the diet analyses, at the 3-year resurvey. All analyses were also adjusted for other factors at recruitment: region of residence at baseline (Scotland and 9 areas in England representing NHS Regional Heath Authorities); education (no qualifications, any qualification)^21^; area deprivation (tertiles based on the Townsend Index^22^); height (<155, 155–164, 165+ cm); smoking (never, past, current <10, 10–19, 20+ cigarettes per day); alcohol consumption (0, <7, 7–14, 15+ drinks per week); and use of menopausal hormones (never, past, current).

Analyses of the associations of dementia with BMI and with physical inactivity were mutually adjusted but were not adjusted for caloric intake because this was measured 3 years later. Analyses of the associations of dementia with diet were adjusted for physical inactivity but not for BMI because BMI could lie on the causal pathway. To keep the numbers analyzed constant, the small number of women with missing data for any adjustment variable (<2% for each variable) were assigned to a separate category for that variable. Because of possible biases associated with poor health at baseline, sensitivity analyses were done that were restricted to the 480,606 women who reported at the 3-year resurvey that they were in good or excellent health.

Because stroke predisposes to dementia,^23^ an analysis was done excluding women with self-reported stroke at baseline and censoring women on the date of any hospital admission with cerebrovascular disease or transient cerebral ischemia (ICD-10 I60-69 or G45).

Analyses used Stata 15.1 (StataCorp, College Station, TX); figures were drawn in R.^24^

### Data availability

Anonymized data used here can be shared by request to the investigators and to the providers of follow-up data (e.g., NHS Digital) from any qualified investigator. The Million Women Study Data Access Policy can be viewed at millionwomenstudy.org/data_access.

## Results

The 1,136,846 women included in the analyses of BMI, physical inactivity, and dementia detection rates were, on average, 56 (SD 5) years of age at baseline. As expected, obesity and physical inactivity were associated, and both were associated with deprivation and lack of educational qualifications (table). Among the 610,069 women included in the dietary analyses, low caloric intake was also associated with deprivation and lack of educational qualifications.

**Table:**
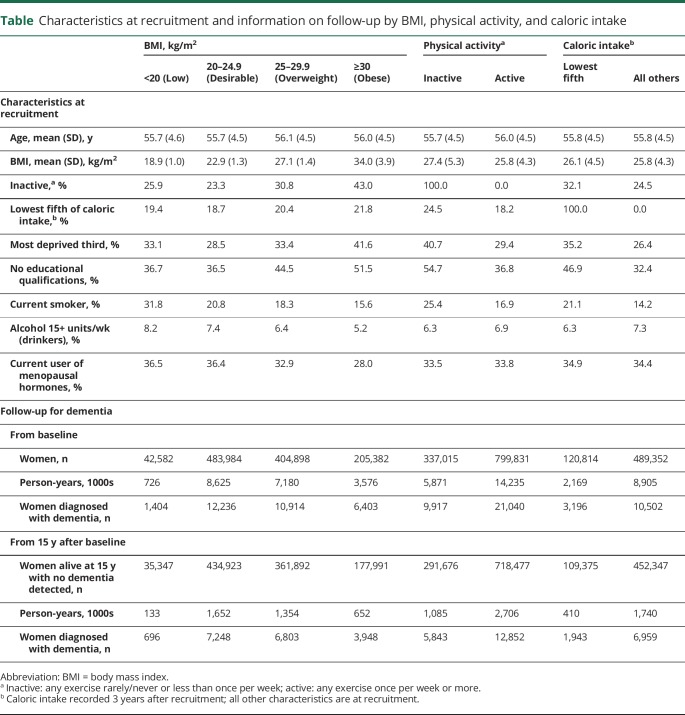
Characteristics at recruitment and information on follow-up by BMI, physical activity, and caloric intake

The entire cohort was followed up for a mean of 18 (SD 3) years to a mean age of 74 (SD 5) years. During that period, 30,957 women had ≥1 hospital admissions with dementia mentioned, of which only the first is analyzed. During the first 5 years of follow-up, 478 women had their first hospital admission with mention of dementia; during follow-up years 5 to 9, a further 2,410 did so; during follow-up years 10 to 14, a further 9,374 did so; and during follow-up years 15+ (mainly years 15–19), a further 18,695 did so, at a mean age of 77 (SD 4) years. At year 15 after study entry, 89% of all participants were still alive with no hospital record mentioning dementia and were still being followed up for details of any subsequent hospital admissions or deaths (table). Apart from having become older, their average characteristics were not materially different from those of the entire population at baseline, and all analyses of dementia detection rates during follow-up years 15+ (mainly years 15–19) are based only on these survivors with no record of dementia before follow-up year 15 (data available from Dryad, table 2, doi.org/10.5061/dryad.3s755j8).

**Figure 1:**
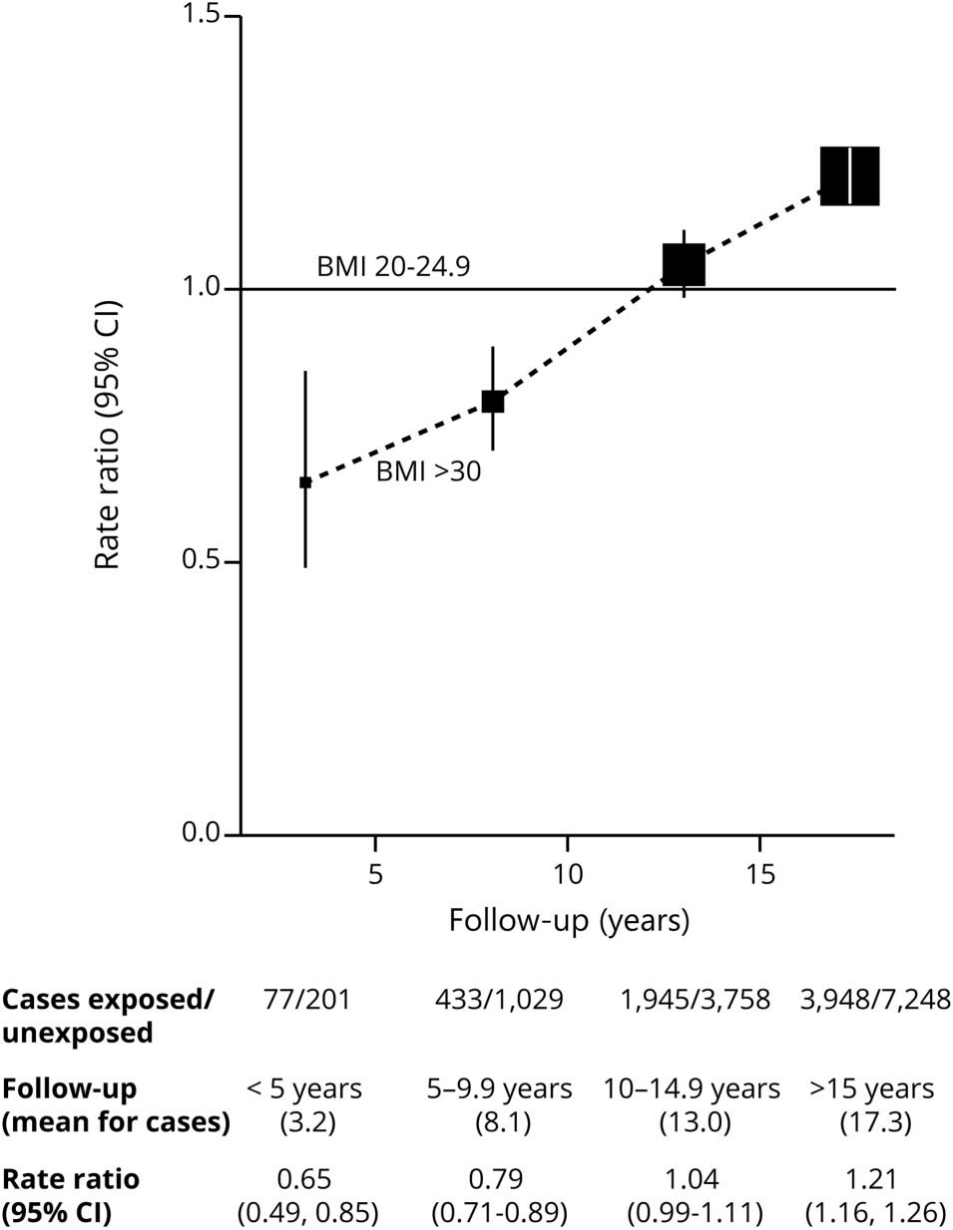
Dementia detection rate ratio by period of follow-up for obese vs desirable BMI (30+ vs 20–24.9 kg/m^2^) Stratified by year of birth and year reporting exposure and adjusted for region of residence, educational qualifications, area deprivation, height, smoking, alcohol consumption, use of menopausal hormones, and physical activity. BMI = body mass index; CI = confidence interval.

The dementia detection RRs among women who had been obese at study entry compared to women who had been of desirable BMI (20–24.9 kg/m^2^) are shown separately in figure 1 for follow-up years <5, 5 to 9, 10 to 14, and 15+ (mostly years 15–19, mean year 17.3 for the cases). During the first 5 years of follow-up, the dementia detection RR for obese vs desirable was only about two-thirds (RR 0.65, 95% CI 0.49–0.85, *p* < 0.0001). During follow-up years 15+, however, the dementia detection rate was significantly higher in women who had been obese than in those who had been of desirable BMI (RR 1.21, 95% CI 1.16–1.26, *p* < 0.0001).

**Figure 2:**
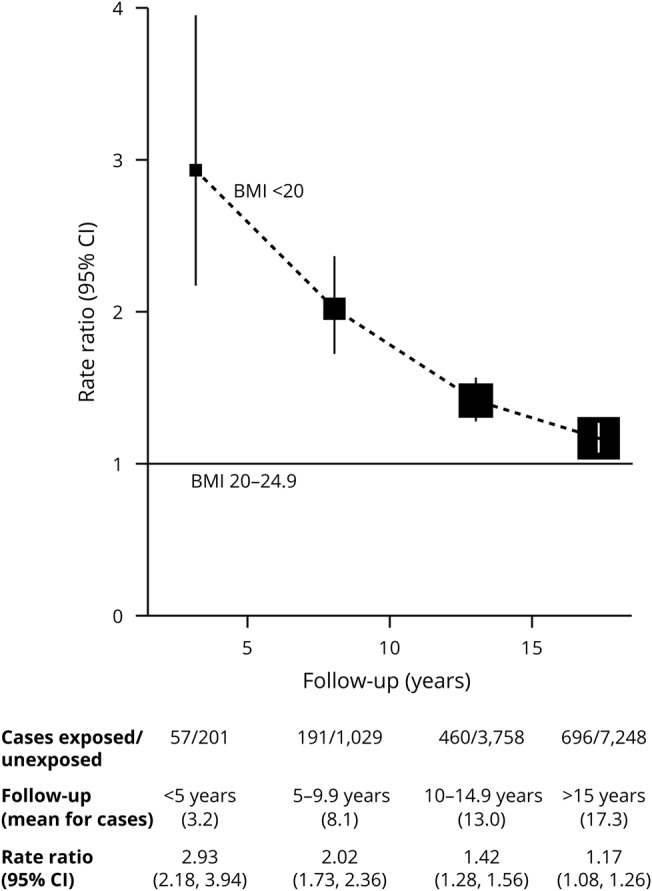
Dementia detection rate ratio by period of follow-up for low vs desirable BMI (<20 vs 20–24.9 kg/m^2^) Stratified by year of birth and year reporting exposure and adjusted for region of residence, educational qualifications, area deprivation, height, smoking, alcohol consumption, use of menopausal hormones, and physical activity. BMI = body mass index; CI = confidence interval.

Comparing low BMI (<20 kg/m^2^) vs desirable BMI (figure 2), the dementia detection RR was almost 3 during the first 5 years of follow-up (RR 2.93, 95% CI 2.18–3.94, *p* < 0.0001), but it declined substantially as the duration of follow-up increased. By follow-up years 15+, it had fallen greatly, although it was still significant (RR 1.17, 95% CI 1.08–1.26, *p* < 0.0001).

**Figure 3:**
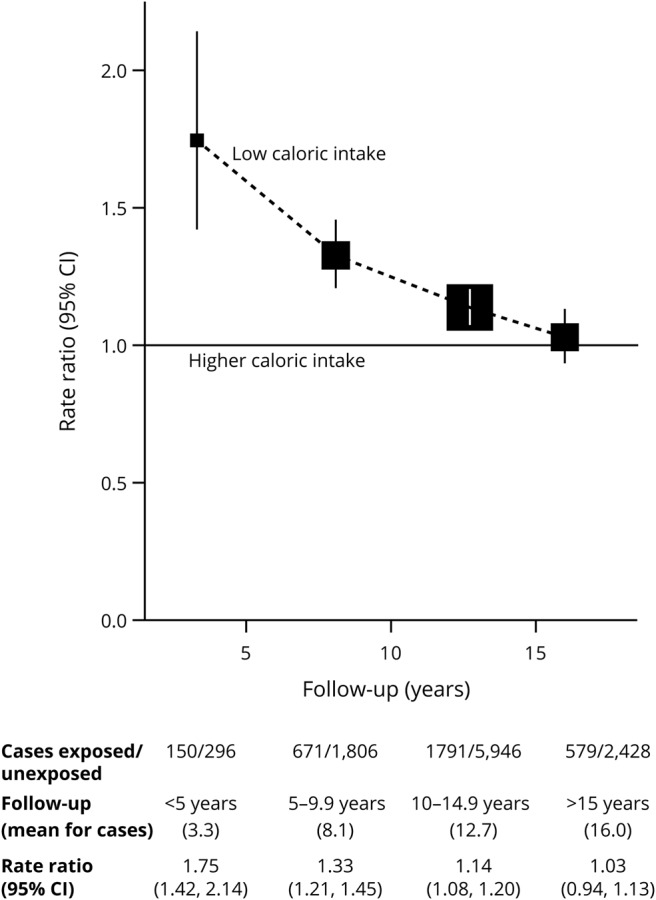
Dementia detection rate ratio by period of follow-up for those with the lowest fifth of caloric intake vs higher intakes Stratified by year of birth and year reporting exposure and adjusted for region of residence, educational qualifications, area deprivation, height, smoking, alcohol consumption, use of menopausal hormones, and physical activity. CI = confidence interval.

The dementia detection RRs comparing women in the lowest fifth of total caloric intake vs all women with higher caloric intakes are shown in figure 3, subdivided by period of follow-up since caloric intake was assessed. Because this assessment was ≈3 years after BMI was recorded, the potential duration of follow-up is ≈3 years shorter for caloric intake than for BMI, but still for the caloric intake analyses, 3,007 cases of dementia were first detected during follow-up years 15+ (mean year 16.0 for the cases). Low caloric intake at the time of the dietary survey was associated with an elevated RR during the first 5 years of follow-up (RR 1.75, 95% CI 1.42–2.14, *p* < 0.0001), but the RR decreased steeply with duration of follow-up, and by follow-up years 15+, no apparent excess remained (RR 1.03, 95% CI 0.94–1.13). During the first decade of follow-up, there was little apparent dependence of dementia detection rates on caloric intake across the top four-fifths of intake, and during the second decade, there was none (data not shown).

For other indices of poor diet (low protein intake as percent of total caloric intake, high free sugar as percent of total caloric intake, low Mediterranean diet score, and low World Health Organization healthy diet indicator), the patterns over time were similar to those seen in figure 3, with RRs of 1.3 to 1.9 during the first 5 years of follow-up, but with the excess RR declining over time and RRs of ≈1.0 during follow-up years 15+ (data available from Dryad, figure 7, doi.org/10.5061/dryad.3s755j8).

**Figure 4:**
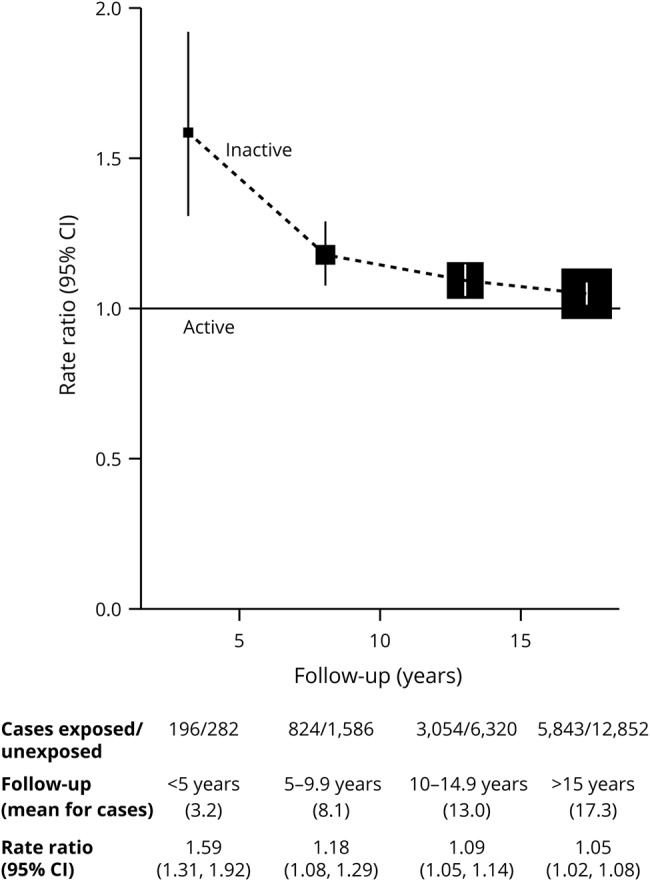
Dementia detection rate ratio by period of follow-up in inactive vs active women Stratified by year of birth and year reporting exposure and adjusted for region of residence, educational qualifications, area deprivation, height, smoking, alcohol consumption, use of menopausal hormones, and body mass index. CI = confidence interval.

The dementia detection RRs comparing inactive and active women are shown in figure 4 by follow-up duration. During the first 5 years of follow-up, inactive women had an ≈60% excess dementia detection rate (RR 1.59, 95% CI 1.31–1.92, *p* < 0.0001), but again, the association weakened over time, remaining significant but approaching null after 15+ years of follow-up (RR 1.05, 95% CI 1.02–1.08, *p* = 0.004). These analyses were adjusted for BMI, but this made little difference (e.g., decreasing the RR for dementia detection during follow-up years 15+ from 1.07 to 1.05).

Sensitivity analyses restricted attention to the 480,606 women who had at the time of the dietary survey rated their health as good or excellent. The short-term RRs tended to be slightly more extreme in these women than in all women, whereas the long-term RRs were similar (data available from Dryad, figure 8, doi.org/10.5061/dryad.3s755j8).

**Figure 5:**
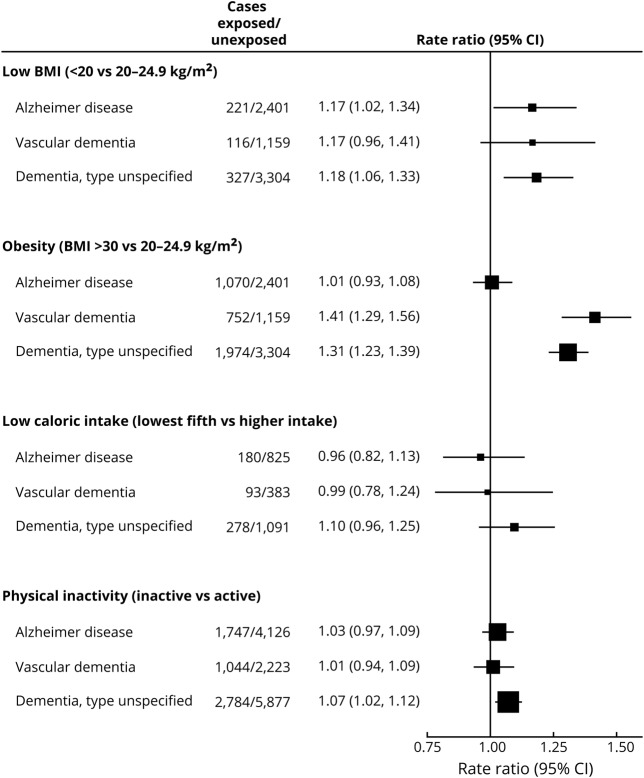
Dementia detection rate ratio after 15+ years of follow-up by hospital-specified type of dementia Stratified by year of birth and year reporting exposure and adjusted for region of residence, educational qualifications, area deprivation, height, smoking, alcohol consumption, and use of menopausal hormones. For body mass index (BMI) and caloric intake analyses, also adjusted for physical activity. For physical activity analysis, also adjusted for BMI. CI = confidence interval.

Both Alzheimer disease and vascular disease can result in dementia and could well have importantly different causes. Figure 5 subdivides the findings for each of the 4 factors examined here by whether the first hospital record of dementia attributed it only to Alzheimer disease (n = 5,873), only to vascular disease (n = 3,267), or to an unspecified disease (n = 8,661); analyses are restricted to follow-up years 15+ because this is when the RRs should have been least distorted by reverse causality. Of the 4 factors, only for obesity was there clear variation between the RRs (obese vs desirable BMI) for what was recorded as Alzheimer disease and for what was recorded as vascular dementia in the first hospital admission that mentioned any type of dementia. During follow-up years 15+, baseline obesity was strongly associated with vascular dementia (RR 1.41, 95% CI 1.29–1.56, *p* < 0.0001) but not with Alzheimer disease (RR 1.01, 95% CI 0.93–1.08, *p* = 0.87, *p* < 0.0001 for heterogeneity between RRs). No confounding factors were identified as having any material effect on these obesity RRs (data available from Dryad, table 3, doi.org/10.5061/dryad.3s755j8). Figure 5 also shows that in about half of the first hospital records that mentioned dementia, the type was unspecified. In many cases when it was specified, the differential diagnosis between vascular dementia and Alzheimer disease would have been uncertain.

During follow-up, 13,181 women had a hospital admission for cerebrovascular disease. Censoring these women on the date of diagnosis of cerebrovascular disease had little effect on the dementia detection RRs during follow-up years 15+ (data available from Dryad, figure 9, doi.org/10.5061/dryad.3s755j8). Likewise, inclusion of an additional 1726 cases whose first mention of dementia was on the death certificate had no material effect on the main findings because far larger numbers had been detected from hospital records (data available from Dryad, figure 10, doi.org/10.5061/dryad.3s755j8).

**Figure 6:**
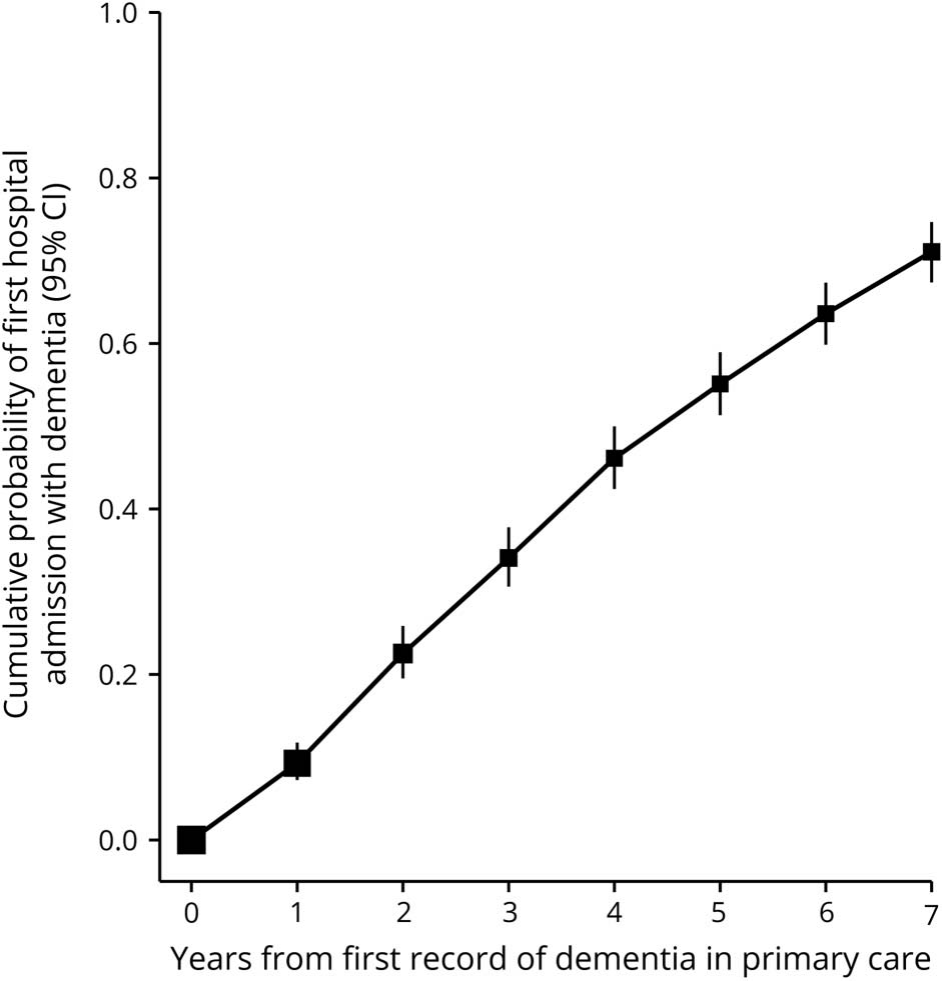
Probability of first admission to hospital with dementia by time since first mention of dementia in primary care CI = confidence interval.

For 76,301 women, some primary care records were provided by linkage to the CPRD. Figure 6 shows that for those with a CPRD primary care record of dementia (some of whom might already have had a hospital record of dementia), the median time from the CPRD record to the next such hospital record was 4 (interquartile range 2–7) years (data available from Dryad, additional Methods 3, doi.org/10.5061/dryad.3s755j8).

## Discussion

During about 2 decades of follow-up, >30,000 participants had a first hospital admission with mention of dementia, most during follow-up years 15+. The gradual preclinical development of dementia can itself cause inactivity, changes in eating patterns, and weight loss, which could distort the baseline data. Perhaps because of this, the dementia detection rates during the first decade of follow-up were not positively associated with baseline obesity and were instead positively associated with baseline low BMI, low caloric intake, and physical inactivity. The latter 3 associations weakened substantially over time, however, and approached null after ≈15 years of follow-up. In contrast, a positive association between baseline obesity and dementia emerged only during the second decade of follow-up, and during follow-up years 15+, baseline obesity was associated with a substantial excess dementia detection rate, at least for vascular dementia.

The average age was 56 years at recruitment and 74 years at the end of follow-up. All participants were registered with the NHS and had a unique NHS number, so follow-up through electronic linkage to NHS databases was virtually complete; only 1.2% were lost to follow-up, mainly because they had emigrated from the United Kingdom and from then on were no longer registered with the NHS. The analyses included those who died or were lost to follow-up until the dates when this occurred, thus allowing fully for competing risks of death. After 15 years of follow-up, 89% were still alive and still being followed up with no hospital record that had mentioned dementia, and (apart from being older) their characteristics at year 15 were similar to those of the entire cohort at baseline. No major secular changes in BMI, diet, or physical activity occurred in this population,^7,13,15^ and confounding by other possible risk factors appeared to have little effect on the findings.

The large differences between the results during the first few years of follow-up and those during follow-up years 15+ suggest that the findings during the first decade of follow-up were substantially distorted by reverse causality, i.e., by preclinical dementia having already, at the time of the baseline data collection, appreciably reduced caloric intake, BMI, and physical activity. Distortion of the baseline information by the effects of preclinical disease should on average have had a greater effect on women whose dementia was detected during the first decade of follow-up than on those whose dementia was detected during the second decade and may well have had little effect on the baseline characteristics of the large number whose dementia was detected during follow-up years 15+. If so, then the associations of low caloric intake, low BMI, and inactivity with dementia detection rates during the first decade of follow-up are not good evidence that any of these 3 factors actually cause dementia, whereas the association of obesity with dementia during follow-up years 15+ does suggest that obesity or some close correlate of obesity causes an increased risk of developing some major type of dementia.

For BMI, our short-term findings are broadly consistent with published evidence from the many prospective studies with short-term follow-up.^5,6^ Long-term associations have been reported in relatively few studies, with inconsistencies between their reported findings.^5,6,25^ Consistent with the present study, the only individual-participant-data meta-analysis of prospective studies reported an inverse association with BMI during the first 20 years of follow-up that reversed after 20 years, so that baseline obesity was associated with an increased risk of dementia developing among those still being followed up >20 years later.^6^ A smaller meta-analysis of published studies reported that, with long-term follow-up, dementia risk was associated both with low BMI at baseline and with obesity at baseline.^5^ In contrast, a UK study based on CPRD primary care records reported decreased risks with obesity that persisted for ≥15 years, although it was not clear whether more recent BMI measurements had in some cases been extrapolated backward.^25^

For obesity, the RR increased with follow-up time, and there was a clear excess of dementia detection during follow-up years 15+. This excess was highly significant and greater for hospital admissions coded as vascular dementia than for hospital admissions coded as Alzheimer disease. This difference between the apparent associations of obesity with the 2 different types of dementia suggests that the association of obesity with vascular dementia is likely to be causal, i.e., that women who are obese in midlife are more likely than otherwise similar women who are of desirable weight to develop dementia some years later. Given the effects in high-income countries of midlife obesity on blood pressure, diabetes mellitus, and occlusive stroke, it is plausible that midlife obesity could also increase the incidence of vascular dementia (and, more generally, that some other factors that predispose to occlusive stroke may, even in the absence of a definite major stroke, also predispose to vascular dementia).

For low caloric intake and physical inactivity, the short-term excess risks in this study are consistent with previous systematic reviews, which are dominated by studies with short-term follow-up.^8–10,26–29^ A recent study with a mean follow-up of 26 years^1^ that was not included in those meta-analyses found no association of dementia risk with physical activity, in line with our long-term findings. The excess dementia detection rates associated with low caloric intake and physical inactivity during the first decade of follow-up that decline with follow-up duration, becoming close to null during follow-up years 15+, are consistent with reports that appreciable weight loss and reduced physical activity can precede the clinical diagnosis of dementia by about a decade^1–4^ and suggest that the short-term associations could well be largely or wholly the consequence of preclinical dementia.

Important strengths of this population-based study are the inclusion of ≈1 in 4 of all UK women born in 1935 to 1950 and the virtually complete long-term follow-up for hospital records that mention dementia over ≈2 decades. The main weakness is that the chief endpoint is a hospital admission with mention of dementia (about half with specific mention of Alzheimer disease or of vascular dementia and about half without). In this cohort, however, <5% of the hospital diagnoses of dementia could not be confirmed in primary care records,^20^ and in England, half of those with a diagnosis of dementia in primary care will within the next 4 years have a hospital admission with mention of dementia. Comparison of NHS hospital diagnoses and expert clinical adjudication showed an overall positive predictive value of 87% for hospital diagnoses of any dementia, with somewhat lower values for the separate endpoints of Alzheimer disease and vascular dementia.^30^ While not all women with dementia diagnosed in primary care are admitted to hospital, only 0.1% of a randomly selected subset of women with no hospital record of the disease in this cohort were reported on inquiry to their primary care physician to have dementia.^20^ Hence, the large majority of women categorized as having dementia and the large majority categorized as not having dementia were correctly classified. Some misclassification is inevitable, which would be expected to dilute any real associations.

The short-term associations of dementia with physical inactivity, low caloric intake, and low BMI are likely to be mainly due to preclinical dementia affecting behavior and weight loss. In this population, midlife obesity is the only factor examined that is likely to be causally related to dementia, perhaps chiefly through its effects on vascular disease.

## Glossary

BMI: body mass index
CI: confidence interval
CPRD: Clinical Practice Research Datalink
ICD-10: *International Classification of Diseases, 10th Revision*
NHS: National Health Service
RR: rate ratio

## References

[1] Sabia S, Dugravot A, Dartigues JF, et al. Physical activity, cognitive decline, and risk of dementia: 28 year follow-up of Whitehall II cohort study. BMJ 2017;357:j2709. 28642251 10.1136/bmj.j2709 PMC5480222

[2] Knopman DS, Edland SD, Cha RH, Petersen RC, Rocca WA. Incident dementia in women is preceded by weight loss by at least a decade. Neurology 2007;69:739–746. 17709705 10.1212/01.wnl.0000267661.65586.33

[3] Stewart R, Masaki K, Xue QL, et al. A 32-year prospective study of change in body weight and incident dementia: the Honolulu-Asia Aging Study. Arch Neurol 2005;62:55–60. 15642850 10.1001/archneur.62.1.55

[4] Singh-Manoux A, Dugravot A, Shipley M, et al. Obesity trajectories and risk of dementia: 28 years of follow-up in the Whitehall II Study. Alzheimers Dement 2018;14:178–186. 28943197 10.1016/j.jalz.2017.06.2637 PMC5805839

[5] Albanese E, Launer LJ, Egger M, et al. Body mass index in midlife and dementia: systematic review and meta-regression analysis of 589,649 men and women followed in longitudinal studies. Alzheimers Dement (Amst) 2017;8:165–178. 28761927 10.1016/j.dadm.2017.05.007 PMC5520956

[6] Kivimaki M, Luukkonen R, Batty GD, et al. Body mass index and risk of dementia: analysis of individual-level data from 1.3 million individuals. Alzheimers Dement 2018;14:601–609. 29169013 10.1016/j.jalz.2017.09.016 PMC5948099

[7] Wright FL, Green J, Reeves G, Beral V, Cairns BJ; Million Women Study Collaborators. Validity over time of self-reported anthropometric variables during follow-up of a large cohort of UK women. BMC Med Res Methodol 2015;15:81. 26450616 10.1186/s12874-015-0075-1 PMC4599695

[8] Blondell SJ, Hammersley-Mather R, Veerman JL. Does physical activity prevent cognitive decline and dementia? A systematic review and meta-analysis of longitudinal studies. BMC Public Health 2014;14:510. 24885250 10.1186/1471-2458-14-510 PMC4064273

[9] Cao L, Tan L, Wang HF, et al. Dietary patterns and risk of dementia: a systematic review and meta-analysis of cohort studies. Mol Neurobiol 2016;53:6144–6154. 26553347 10.1007/s12035-015-9516-4

[10] Xu W, Wang HF, Wan Y, Tan CC, Yu JT, Tan L. Leisure time physical activity and dementia risk: a dose-response meta-analysis of prospective studies. BMJ Open 2017;7:e014706. 10.1136/bmjopen-2016-014706 PMC5665289 29061599

[11] Global BMI Mortality Collaboration (Writing Committee), Di Angelantonio E, Bhupathiraju S, Wormser D, et al. Body-mass index and all-cause mortality: individual-participant-data meta-analysis of 239 prospective studies in four continents. Lancet 2016;388:776–786. 27423262 10.1016/S0140-6736(16)30175-1 PMC4995441

[12] Green J, Reeves GK, Floud S, et al. Cohort profile: the Million Women Study. Int J Epidemiol 2019;48:28–29e. 29873753 10.1093/ije/dyy065 PMC6380310

[13] Armstrong ME, Cairns BJ, Green J, Reeves GK, Beral V; Million Women Study Collaborators. Reported frequency of physical activity in a large epidemiological study: relationship to specific activities and repeatability over time. BMC Med Res Methodol 2011;11:97. 21831330 10.1186/1471-2288-11-97 PMC3145605

[14] Roddam AW, Spencer E, Banks E, et al. Reproducibility of a short semi-quantitative food group questionnaire and its performance in estimating nutrient intake compared with a 7-day diet diary in the Million Women Study. Public Health Nutr 2005;8:201–213. 15877913 10.1079/phn2004676

[15] Key T, Balkwill A, Bradbury KE, et al. Foods, macronutrients and breast cancer risk in postmenopausal women: a large UK cohort. Int J Epidemiol 2019;48:489–500. 30412247 10.1093/ije/dyy238 PMC6469308

[16] NHS Digital [online]. Available at: digital.nhs.uk/. Accessed April 27, 2018.

[17] NHSCR Scotland [online]. Available at: www.nrscotland.gov.uk/statistics-and-data/nhs-central-register. Accessed August 29, 2018.

[18] World Health Organization. International Statistical Classification of Diseases and Related Health Problems, 10th revision. 2nd ed. Geneva: World Health Organization; 2004.

[19] Herrett E, Gallagher AM, Bhaskaran K, et al. Data resource profile: Clinical Practice Research Datalink (CPRD). Int J Epidemiol 2015;44:827–836. 26050254 10.1093/ije/dyv098 PMC4521131

[20] Brown A, Kirichek O, Balkwill A, et al. Comparison of dementia recorded in routinely collected hospital admission data in England with dementia recorded in primary care. Emerg Themes Epidemiol 2016;13:11. 27800007 10.1186/s12982-016-0053-z PMC5084368

[21] Floud S, Balkwill A, Moser K, et al. The role of health-related behavioural factors in accounting for inequalities in coronary heart disease risk by education and area deprivation: prospective study of 1.2 million UK women. BMC Med 2016;14:145. 27733163 10.1186/s12916-016-0687-2 PMC5062936

[22] Townsend P, Beattie A, Phillimore P. Health and Deprivation: Inequality and the North. London: Croom Helm; 1988.

[23] Savva GM, Stephan BC; Alzheimer's Society Vascular Dementia Systematic Review Group. Epidemiological studies of the effect of stroke on incident dementia: a systematic review. Stroke 2010;41:e41–46. 19910553 10.1161/STROKEAHA.109.559880

[24] R Core Team. R: A language and environment for statistical computing: R foundation for statistical computing. Vienna. 2013. Available at: www.R-project.org/. Accessed April 25, 2019.

[25] Qizilbash N, Gregson J, Johnson ME, et al. BMI and risk of dementia in two million people over two decades: a retrospective cohort study. Lancet Diabetes Endocrinol 2015;3:431–436. 25866264 10.1016/S2213-8587(15)00033-9

[26] Lourida I, Soni M, Thompson-Coon J, et al. Mediterranean diet, cognitive function, and dementia: a systematic review. Epidemiology 2013;24:479–489. 23680940 10.1097/EDE.0b013e3182944410

[27] Solfrizzi V, Custodero C, Lozupone M, et al. Relationships of dietary patterns, foods, and micro- and macronutrients with Alzheimer's disease and late-life cognitive disorders: a systematic review. J Alzheimers Dis 2017;59:815–849. 28697569 10.3233/JAD-170248

[28] van de Rest O, Berendsen AA, Haveman-Nies A, de Groot LC. Dietary patterns, cognitive decline, and dementia: a systematic review. Adv Nutr 2015;6:154–168. 25770254 10.3945/an.114.007617 PMC4352174

[29] Yusufov M, Weyandt LL, Piryatinsky I. Alzheimer's disease and diet: a systematic review. Int J Neurosci 2017;127:161–175. 26887612 10.3109/00207454.2016.1155572

[30] Wilkinson T, Schnier C, Bush K, et al. Identifying dementia outcomes in UK Biobank: a validation study of primary care, hospital admissions and mortality data. Eur J Epidemiol 2019;34:557–565. 30806901 10.1007/s10654-019-00499-1 PMC6497624

